# A Review of Corneal Collagen Cross-linking – Current Trends in Practice Applications

**DOI:** 10.2174/1874364101812010181

**Published:** 2018-07-23

**Authors:** Li Lim, Elizabeth Wen Ling Lim

**Affiliations:** 1 MBBS (Singapore), MMed (Ophth), FRCS(Ed), FAMS (S'pore) Senior Consultant, Corneal and External Eye Disease Service, Singapore National Eye Centre, Singapore, Singapore; 2 Undergraduate medical student, Yong Loo Lin School of Medicine, National University of Singapore, Singapore, Singapore

**Keywords:** Cornea collagen cross-linking, post-LASIK ectasia, Keratoconus, Infective keratitis, UVA, Dresden protocol

## Abstract

**Objective:**

To review the literature on current applications of corneal Collagen Cross-Linking (CXL).

**Methods:**

A review of publications on corneal cross-linking was conducted. This included systemic reviews, randomized controlled clinical trials, cohort studies, case-controlled studies and case series. A summary of the publications is tabulated.

**Results:**

The original indication of riboflavin – Ultraviolet-A (UVA) induced corneal collagen cross-linking is to arrest the progression of keratoconus. Studies show that it is effective in arresting the progression of keratoconus and post-LASIK ectasia with the standard Dresden protocol (epithelium-off). There are also improvements in visual, keratometric and topographic measurements over time. Severe complications of cross-linking are rare. The epithelium-on techniques have less efficacy than the Dresden protocol. Accelerated protocols have variable results, with some studies reporting comparable outcomes to the Dresden protocol while other studies reporting less efficacious outcomes. Cross-linking combined with refractive procedures provide better visual outcome but long term studies are warranted. Cross-linking for the treatment of infective keratitis is a promising new treatment modality. Initial studies show that it is more effective for superficial rather than deep infections and for bacterial rather than fungal infections.

**Conclusions:**

Corneal cross-linking is a procedure with an expanding list of indications from the treatment of corneal ectasias to infective keratitis. While the standard Dresden protocol is established as the gold standard treatment for progressive keratoconus, the more recent protocols may require further refinements, investigative and long-term studies.

## 1 INTRODUCTION

Wollensak, Spoerl and Seiler reported the first clinical study on riboflavin – UVA induced corneal collagen cross-linking procedure for the treatment of progressive keratoconus in adults [1]. Since then, corneal cross-linking has been widely used for the treatment of progressive keratoconus as well as other conditions including post-LASIK ectasia. Corneal cross-linking is also used in conjunction with laser vision correction procedures and intrastromal ring procedures. More recently, it is used to treat infective keratitis. The purpose of this paper is to provide a review of the current trends in practice applications of corneal collagen cross-linking.

## 2 OVERVIEW

Epithelium-off CXL (Dresden protocol)

Epithelium-on CXL

Accelerated CXL

CXL plus (Cross-linking combined with Refractive Procedures for the treatment of Corneal Ectatic Disorders)

Customised CXL

Combined laser *in-situ* keratomileusis(LASIK) and accelerated CXL

Cross-linking for the treatment of Infective Keratitis (PACK-CXL)

Other Applications of CXL

Cross-linking complications

## 3 STANDARD EPITHELIUM–OFF CXL PROTOCOL (DRESDEN PROTOCOL)

Wollensak, Spoerl and Seiler introduced the Dresden protocol that has become the standard procedure of cross-linking today [1]. A documented evidence of progression of the keratoconus should be obtained before performing cross-linking. Studies show that progression may be defined as a more than 1 Diopter (D) increase in Kmax (maximum keratometry) and/or a more than 1D increase in average keratometry and/or refractive astigmatism of more than 1D and/or decrease in pachymetry of more than 10% in the preceding 12-18 months [2-4]. A minimum corneal stromal thickness of 400um is recommended prior to surgery for the safety of the corneal endothelium [5].

The cross-linking procedure is performed under topical anesthesia (eg tetracaine 1%). An epithelial debridement (8-9mm) is performed followed by instillation of riboflavin 0.1% eyedrops for 30 minutes at 2 minute intervals. The riboflavin solution may contain 20% dextran as in the earlier studies [1] or may be dextran free containing hydroxypropyl methylcellulose [6]. The dextran-free riboflavin preparation may reduce the incidence of stromal dehydration during the procedure. At the end of the riboflavin instillation, ultrasound pachymetry is performed at the centre of the cornea and if the corneal thickness is below 400um, hypotonic riboflavin eyedrops are applied until the corneal thickness returns to a minimum of 400 um thickness. The cornea is then irradiated with the Ultraviolet A (UVA) device at 3mW/cm^2^ for 30 minutes with continued instillation of riboflavin eyedrops at 2-minute intervals. After irradiation is completed, topical antibiotics/steroids and bandage lens are applied.

A corneal stromal demarcation line can sometimes be detected at the slit lamp or with the Anterior Segment Optical Coherence Tomography (AS-OCT) and is most apparent in the first 3 months after cross-linking [7]. Keratocyte apoptosis is observed anterior to the demarcation line on confocal microscopy and repopulation of keratocytes occurs after 3 months. The depth of the demarcation line has commonly been interpreted as the depth of cross-linking [8, 9].

## 4 STABILISING ADULT KERATOCONUS AND POST-LASIK ECTASIA (TABLES **1A**, **1B**, **1C** and **2**)

The primary indications for corneal cross-linking are progressive keratoconus in adults and post-LASIK ectasia. Stabilisation of ectasia with up to five years follow-up was reported in 23 eyes that underwent the epithelium-off CXL technique with an average reduction in spherical equivalent refractive error of 1.0D and maximum keratometry (Kmax) of 2.0D [1]. Subsequently, other prospective case cohort studies showed similar results of stabilization of keratoconus and improvements in visual acuity and topography [10-16]. Comparative studies using the fellow eye as control showed stabilization of the treated eye and continued progression in the fellow untreated eye [17].

Wittig-Silva *et al* performed the first randomized controlled trial and found significant flattening of the steepest keratometry and a trend towards better visual acuity with long-term follow-up showing continued flattening up to 4 years after treatment [3, 4]. Long term studies with follow-up ranging from 4 to 7 years also show improvements in visual acuity and corneal topography [18-24]. O’Brart *et al.* reported improvements in topographic and wavefront parameters evident at 1 year and which continue to improve after 7 years [24].

Epithelium-off CXL has also been shown to be effective in stabilizing post-LASIK ectasia with improvements in the visual acuity with more than 2 years follow-up [25-27]. The US based prospective clinical trial for cross-linking showed improvement in visual acuity and maximum keratometry in patients with keratoconus and post-LASIK ectasia [28]. The keratoconus group had more corneal flattening than ectasia patients. A longer-term study by Richoz [29] with a mean follow-up of 25 months showed improved mean corrected visual acuity and mean Kmax.

## 5 PAEDIATRIC KERATOCONUS

Paediatric cases often present with keratoconus that progress more rapidly than adult onset keratoconus. One study showed that 88% of paediatric cases progress over a short period of time [33]. Hence it is not necessary to document progression and treatment of paediatric keratoconus is recommended at the time of presentation. Studies show that there is an initial favorable response with improvements in visual acuity, keratometry and spherical equivalent at one year followup [34, 35]. In the long term, studies show that the keratoconus continue to progress despite the initial response [33]. Studies also show that the epithelium-off technique is to be preferred over transepithelial cross-linking as the latter technique show worsening keratometry values over time [36].

## 6 EPITHELIUM-ON (TRANSEPITHELIAL) CORNEAL CROSS-LINKING (TABLES **3A** and **3B**)

The standard Dresden protocol (epithelium-off) corneal cross-linking is associated with significant postoperative pain and visual recovery is gradual. There are also risks of infection and corneal scarring. Hence, epithelium-on corneal cross-linking was introduced to reduce the issues associated with the standard protocol. However, riboflavin is a hydrophilic molecule making penetration through the intact hydrophobic corneal epithelium difficult. In order to improve epithelial permeability to riboflavin, additives such as benzalkonium chloride, topical aneasthetic, tris-hydroxymethyl aminomethane (trometamol), sodium ethylenediaminetetraacetic acid (EDTA) are included in the riboflavin. Other techniques include increased riboflavin concentration and iontophoresis.

The outcomes for transepithelial cross-linking using riboflavin with trometamol/EDTA are mixed. Some studies report improvement in visual acuity and keratometry measurements [37-39] while other studies report worsening of keratometry measurements [36, 40]. Also the demarcation line was noted to be shallower in the transepithelial group [37].

The majority of randomized clinical trials comparing the standard and transepithelial technique report better outcomes with the standard technique (reduced Kmax), whilst the transepithelial technique had worsening of keratometry measurements (Kmax) [41, 42].

Transepithelial iontophoretic cross-linking involves the application of a small (1mA) negative charge to enhance riboflavin absorption. Clinical studies showed better results than transepithelial cross-linking alone with improved visual acuity and stability of refraction and topography [43, 44]. Iontophoretic transepithelial cross-linking has also been used in the treatment of paediatric keratoconus using the accelerated protocol with favorable outcome (improved visual acuity and stability of refraction and topography) [45, 46].

## 7 ACCELERATED CROSS-LINKING (TABLES **4A** and **4B**)

The Bunsen-Roscoe Law of Reciprocity states that the photochemical biological effect of ultraviolet light is proportional to the total energy dose delivered, regardless of the applied irradiance and time [49]. In the context of UVA cross-linking, for the same energy dose delivered, one could shorten the duration of treatment by applying a higher irradiance power. Laboratory studies show that this law could be applied for corneal cross-linking. Wernli *et al* treated ex vivo porcine eyes with CXL using a total of 5.4J/cm^2^ delivered in a range of irradiances from 3 to 90mW/cm^2^. Significant stiffening was observed in eyes treated with irradiances from 3 to 45mW/cm^2^ [50].

Kanellopoulos reported the first clinical study (randomized prospective contralateral eye study) on accelerated cross-linking using 7mW/cm^2^ irradiation 15 minute protocol (5.4J/cm^2^) and Dresden protocol [51]. Stabilisation of keratoconus was achieved in both groups with a flattening of steep keratometry observed in both groups with no change in the endothelial cell density. Subsequent studies employed higher irradiances and shorter duration times: Gatzioufas *et al* reported preliminary results using 18mW/cm^2^ for 5min at 5.4J/cm^2^ with no complications [52], Tomita reported on a comparative study on accelerated CXL(30mW/cm^2^ for 3 minutes at 5.4J/cm^2^) and CXL(Dresden protocol). No statistical differences were found between the two groups for uncorrected distance visual acuity or corrected distance acuity and significant flattening was observed in average keratometry in both groups at 1 year follow-up [53]. Tomita also compared the demarcation line between CXL and accelerated CXL(30mW/cm^2^ for 3 minutes at 5.4J/cm^2^) and described a mean demarcation line depth of 294.38 +/-60.57 um in the accelerated group and 380.78 +/-54.99 um in the CXL group. The difference was not statistically significant. Kymionis reported a greater depth mean demarcation line (350.75 +/-49.34um) in the Dresden CXL than the accelerated CXL (9mW/cm^2^ for 10 minutes) with mean demarcation line at 288.46 +/-42.37um [54]. However, when the protocol was changed to 9mW/cm^2^ for 14 minutes, there was no difference in corneal stromal demarcation line depth between Dresden CXL and accelerated CXL [55].

Shetty *et al* reported a comparative study of CXL and accelerated CXL in 138 eyes of 138 patients with 1 year follow-up [56]. He reported that the accelerated CXL (9mW/cm^2^ for 10 minutes and 18mW/cm^2^ for 5 minutes) had similar outcomes to standard CXL but the accelerated CXL using 30mW/cm^2^ for 3 minutes was not as efficacious.

The reduced efficacy of the 30mW/cm^2^ treatment is postulated to be due to the depletion of oxygen in these high fluence treatments and pulsed treatments were introduced in an effort to replenish oxygen in the cornea during high fluence treatments. Mazotta *et al* [57] reported a greater reduction of keratometry in pulsed compared to continuous treatment

The treatment protocol of accelerated CXL is still in evolution due to the variability of the outcomes reported. Further long term studies are needed to confirm the comparability of accelerated CXL to CXL.

## 8 CROSS-LINKING COMBINED WITH REFRACTIVE PROCEDURES FOR THE TREATMENT OF CORNEAL ECTASIA (CXL PLUS)

Although CXL is effective in stabilizing keratoconus, in many cases, patients are unable to achieve functional vision after CXL and still require rigid contact lens wear. Hence refractive treatments in combination with CXL (CXL plus) have been introduced to provide patients with better visual acuity.

### 8.1 Photorefractive Keratectomy (PRK) and CXL

Kanellopoulos and Binder reported on the first case of topography-guided PRK performed 1 year after CXL for the treatment of keratoconus showing improvement in visual acuity [67]. Subsequently Kanellopuolos reported that simultaneous treatment (PRK followed by CXL) is more effective than sequential treatment (CXL followed 6 months later by PRK) [68] in the visual rehabilitation of keratoconus. Other studies also confirmed the safety and efficacy of simultaneous topography guided PRK and CXL [69-77]. Some studies advocate the use of mitomycin C 0.02% after laser ablation while others do not.

### 8.2 Transepithelial Phototherapeutic Keratectomy (PTK) and CXL

The removal of corneal epithelium in the CXL procedure is replaced with PTK which not only removes epithelium but also regularises the anterior corneal surface [78]. Kymionis *et al*, in a comparative study showed that epithelial removal using PTK during CXL (Cretan protocol) results in better visual and refractive outcomes than mechanical removal of epithelium [79]. Transepithelial PTK uses the patient’s epithelium as a masking agent. At the apex of the cone, epithelium and the anterior stromal surface is removed resulting in a more regularized anterior corneal surface [78].

### 8.3 CXL and Intrastromal Corneal Ring Segment (ICRS) Implantation

Intrastromal Corneal Ring Segment (ICRS) implantation is currently a treatment option for keratoconus and post-LASIK ectasia [80-82]. However, it does not prevent keratoconus progression and in young patients with progressive keratoconus, CXL may be performed in addition to ICRS to add biomechanical stability. Chan *et al* reported that ICRS (Intacs) with CXL resulted in better keratoconus improvement than Intacs insertion alone [83]. Coskunseven reported that ICRS implantation followed by CXL resulted in greater keratoconus improvement than CXL followed by ICRS [84]. El Awady reported that CXL has an additive effect after Keraring implantation (Mediphacos, Belo Horizonte, Brazil) [85]. Studies on simultaneous transepithelial ICRS – CXL report that CXL has an additive effect on ICRS [86, 87]. Lam *et al*. reported a case of post-LASIK ectasia treated with femtosecond laser-assisted ICRS implantation followed by CXL resulting in stabilization of ectasia and improvement in vision [88].

### 8.4 CXL and Phakic Intraocular Lens Implantation

Several case series report on the safety and efficacy of CXL followed by toric Visian ICL [89-91]. Similar results were obtained with Artiflex lens implantation 6 months after CXL and toric iris-claw lens implantation (Artiflex: Ophtec BV) [92]

## 9 CUSTOMISED CROSS-LINKING

Kanellopoulos first reported on a case of customized high fluence toric application of transepithelial cross-linking which resulted in a reduction in corneal astigmatism(0.8D) and improvement in the uncorrected visual acuity from 20/40 to 20/25 at 6 months followup [93].

Roy and Dupps demonstrated using three dimensional finite element analysis model that there is differential biomechanical weakening in the area of the cone. They concluded that there is greater efficacy of smaller diameter cone-centric treatments for the reduction of corneal curvature [94]. The Mosaic delivery system (KXL II, Avedro Inc.,Waltham, MA, USA) offers customised cross-linking (photorefractive intrastromal cross-linking- PiXL). Initial studies on customised cross-liking report greater corneal regularisation and reduction in maximum keratometry than conventional cross-linking [95-97].

Customised cross-linking has recently been used to correct low degrees of refractive error in a patient without keratoconus. Kanellopoulos first described the preliminary results for low myopic correction [98]. Lim et al reported on the results of PiXL for the treatment of low myopia in a cohort of 14 eyes with a 1 year followup [99]. High fluence UV-A irradiation ranging from 10-15 J/cm was delivered over a 4.5mm central zone. A mean reduction of 0.72 +/-0.43D was noted at 1 year followup. Kanellopoulos reported on the results of PiXL for hyperopia, with a mean correction of +0.85D [100].

## 10 COMBINED LASER *IN-SITU* KERATOMILEUSIS (LASIK) AND ACCELERATED CORNEAL CROSS-LINKING

LASIK, with the creation of a corneal flap and ablation of corneal tissue weakens the biomechanical strength of the cornea and in susceptible eyes, may predispose to post-LASIK ectasia. In order to strengthen the cornea, accelerated corneal cross-linking is performed simultaneously after the LASIK procedure. Studies have reported that combined laser *in-situ* Keratomileusis (LASIK) and accelerated corneal cross-linking may confer additional benefits of early refractive and keratometric stability after LASIK, improving the predictability of refractive outcomes in patients. The indications are high myopia corrections, hyperopic corrections, patients with lower residual stromal bed thickness and patients with thin corneas.

LASIK in patients with high myopia has a higher incidence of refractive regression [101, 102]. Hence the use of simultaneous accelerated CXL and LASIK to stabilize the patient’s refraction may be useful particularly in patients with high myopia. In a prospective study comparing 73 LASIK Xtra eyes and 82 LASIK only eyes, Kanellopoulos *et al* found that 90.4% of LASIK Xtra eyes had UDVA of 20/20 or better as compared to 85.4% of LASIK only eyes at post-operative month 12 (p = 0.042) [103]. Similar findings were also shown in another prospective study comparing LASIK Xtra in one eye and LASIK only in the fellow eye over a 12-month period [104].

Kanellopoulos *et al* also reported that corneal keratometry measurements were stable for LASIK Xtra eyes and slightly regressing in LASIK only eyes (p = 0.039) [103]. Subsequently, Kanellopoulos also reported a statistically significant reduction in regression in a 2 year analysis of LASIK-CXL for high myopia compared to the LASIK only group [105]. LASIK and accelerated cross-linking for hyperopia also showed better refractive stability and less regression than LASIK only [106]. Another study by Kanellopoulos found significantly less epithelial thickening in the LASIK and accelerated CXL group compared to the LASIK only group. This could possibly explain the differences in the refractive stability between the 2 groups [107]. LASIK and accelerated CXL has been shown to be comparable to LASIK only in terms of safety, as evidenced by similar loss of corrected visual acuity in both groups [103, 105].

Studies on LASIK and accelerated cross-linking report the use of different levels of UV irradiance, energy levels and illumination times [104, 105, 108, 109]. The energy levels vary from 1.8J/cm^2^ to 5.4J/cm^2^. It is postulated energy settings may be lower (1.8J/cm^2^) than conventional cross-linking treatment for keratoconus (5.4J/cm^2^) since eyes undergoing LASIK and accelerated cross-linking are normal eyes.

One of the goals of performing LASIK with accelerated CXL is reducing the risk of post-LASIK ectasia. A review of the literature of eyes that had undergone LASIK and accelerated cross-linking with at least 2 years follow-up showed no report of post-LASIK ectasia supporting the claim that LASIK with accelerated CXL may prevent post-LASIK ectasia [110]. However, post-LASIK ectasia has been shown to develop as long as 5 to 10 years postoperatively. Hence these reports are not sufficient to make this conclusion and further long term studies are warranted.

## 11 CROSS-LINKING FOR THE TREATMENT OF INFECTIVE KERATITIS (PACK-CXL) (TABLE **5**)

Infectious keratitis is a serious, sight-threatening condition that can result from bacterial, viral, fungal or protozoal infection. Standard treatment for infectious keratitis involves both systemic and topical antimicrobial therapy. However, the effectiveness of this treatment depends on microbial sensitivity to the drug as well as severity of the disease process. Infections not responding to antimicrobial therapy may require therapeutic keratoplasty (lamellar or penetrating). Corneal Collagen cross-Linking (CXL), or Photo Activated Chromophore for Keratitis (PACK-CXL) has been investigated as a possible alternative treatment for infectious keratitis. CXL treatment stiffens the corneal stroma through the effect of photo-activated riboflavin on collagen fibers. This makes the cornea more resistant to enzymatic degradation by microbes, thus reducing the progression of corneal melting [111, 112]. Also, in CXL, riboflavin enters an excited state and reacts with ambient oxygen to create Reactive Oxygen Species (ROS). These ROS cause cell death by damaging intracellular components. Microorganisms are also killed by ROS damage to microbe DNA and cytoplasmic membrane, resulting in leakage of cellular contents and inactivation of enzymes and membrane transport systems [113].

Iseli *et al* first reported the use of PACK-CXL in infectious keratitis in 2008 [114]. Progression of corneal melting was successfully halted, with emergency keratoplasty not required in any of the cases. Subsequently, other case series report that PACK-CXL is effective in treating infectious keratitis caused by different organisms [115-124]. CXL is contraindicated in eyes with previous herpes simplex [125]. It has been shown to be more effective for superficial rather than deep infections [125-127] and for bacterial rather than fungal infections [125]. Makdoumi *et al.* is the first to report treating bacterial keratitis with only PACK-CXL and no antibiotics [120]. Results were successful and all eyes responded to the treatment, with only 2 eyes requiring additional antibiotics and 1 eye requiring an amniotic membrane transplant.

However, comparative clinical trials show that PACK-CXL with antimicrobial treatment had similar results as the control group (only antimicrobial treatment) in terms of healing time and corrected visual acuity [128-130]. The PACK-CXL group had a bigger corneal ulceration width and length [128] and a higher risk of perforation was noted for deep fungal keratitis [130].

## 12 OTHER APPLICATIONS OF CROSS-LINKING

Corneal cross-linking can be employed to prevent further progression in pellucid marginal degeneration(PMD) which is considered a variant of keratoconus. Several studies report on its safety and efficacy [133, 134] [135]. Additionally Kymionis performed simultaneous photorefractive keratectomy and CXL in a both eyes of a patient with PMD resulting in significant improvement in the corneal topography measurements and visual acuity [136].

Corneal cross-linking has also been used as a treatment for pain relief in bullous keratopathy. Sharma et al reported on cross-linking treatment in 50 eyes with bullous keratopathy and concluded that the pain relief achieved was temporary with corneal bullae recurring in 44% of the cases. No long term improvement in visual acuity was seen [137]. Kozobolis et al reported on CXL as an adjunctive treatment for patients with combined bullous keratopathy and infective keratitis [138].

Mukherjee et al performed an animal model evaluation of cross-linking donor corneas for penetrating keratoplasty and concluded that it reduces intraoperative induced astigmatism and aberrations in an animal model [139]. Ting *et al* [140] conducted a randomised controlled trial to investigate whether donor corneas pre-treated with cross-linking reduced myopic refractive errors for keratoconic eyes after penetrating keratoplasty. At 3 years followup, they found significantly improved corrected visual acuity, reduced Kmax and keratometric astigmatism in the CXL treated group.

Crosslinked corneal tissue has been shown to have stiffer biomechanical properties and to be more resistant to degradation by collagenolytic enzymes. Robert et al reported on cross-linking of the Boston keratoprosthesis donor carrier to prevent corneal melting in a patient with post KPro corneal melt. The patient maintained his visual acuity and showed no evidence of corneal thinning or melt in the first postoperative year [141].

## 13 CROSS-LINKING COMPLICATIONS

Complications of corneal cross-linking include corneal haze, corneal scarring, infective keratitis, sterile infiltrates, delayed epithelial healing, failure of treatment, excessive corneal flattening with hyperopic shift and endothelial failure [142]

Anterior corneal haze occurs frequently and usually appears 1-2 months after cross-linking. It is usually transient and clears by 6 to 12 months [142]. Permanent stromal scarring [143] may occur and the incidence has been reported to be as high as 8.6% in one series [144]. It may also be more prevalent in eyes receiving simultaneous PRK followed by CXL [145]. Infective keratitis after cross-linking is rare. Shetty et al reported an incidence of 0.0017% (4 out of 2350 patients) with all 4 cases treated with the epithelium-off technique [146]. Sterile infiltrates present in the early postoperative period (days to weeks) and usually resolve with topical steroid medication [147]. Other uncommon complications include corneal melting associated with atopic eye disease [148] and reactivation of herpetic keratitis [149] Cross-linking should be avoided in patients with previous herpetic eye disease and atopic eye disease should be controlled prior to CXL.

Long-term studies show that progression of keratoconus after cross-linking may occur in about 8% of cases [23, 24, 150]. Hence it is necessary to counsel patients preoperatively about the various potential side-effects and also about the failure rate of the procedure.

Corneal endothelial damage may occur if the safety limits regarding corneal thickness to prevent endothelial toxicity are not adhered to. Sharma *et al* reported a 1.4% incidence of persistent endothelial failure in 350 eyes treated with the standard epithelium-off protocol although the safety limit of corneal thicknesses of greater than 400um (epithelium-off) were adhered to [151]. This could be due to intraoperative stromal dehydration resulting in stromal thinning, lack of homogeneity and focusing/alignment issues of the UV devices.

Although limbal stem cell damage after CXL has been shown in cadaveric eyes [152], long term studies show no evidence of limbal dysfunction [18, 19, 21].

## CONCLUSION

Corneal cross-linking is a unique procedure with an expanding list of indications from the treatment of corneal ectasia to infective keratitis. While the standard Dresden protocol is established as the gold standard treatment for progressive keratoconus, the more recent protocols may require further refinements, investigations and long-term studies. New indications and treatment protocols are also being developed and we look forward to these treatments in the future.

## Figures and Tables

**Table 1A:** Summary of outcomes for standard epithelium-off cross-linking. (Prospective randomised studies) (3mW/cm^2^ UV-A exposure, 5.4J/cm^2^).

**Study**	**Study design/ Indication**	**No. of Eyes**	**Follow-up, Months**	**Criteria for Progression**	**UV device/ Riboflavin**	**Outcome**					
–	–	–	–	–	–	**Overall**	**Pre-op K (D)**	**ΔK (D)**	**ΔUCVA**	**ΔBCVA**	**Δ Refraction (D)**
Henriquez *et al*, 2011 [14]	Prospective, randomised/ Keratoconus	20; 10 treated, 10 FE control	12	1. ↑ Kmax of 1.00 D/1 year 2. ↓ visual acuity 3. New contact lens fitting > once in 2 years	UV-X 1000; IROC AG/ Riboflavin 0.1% w dextran	Improved UCVA. Reduction in mean-K, max-K and min-K, mean SE, anterior and posterior elevation values.	-	Mean Max K (treated): -2.66 (P = 0.04)	Treated: From 1.18 ± 0.80 to 0.46 ± 0.36 (LogMAR) (P < 0.001)	Treated: From 0.20 ± 0.18 to 0.09 ± 0.09 (LogMAR) (P = 0.06)	Mean SE (treated): -2.25 (P = 0.01)
Hersh *et al*, 2011 [28]	Prospective randomised/ Keratoconus and post-laser ectasia	142; 71 treated, 41 sham control, 30 FE control	12	↑ 1D in steepest K, 1D cyl, 0.5D MRSE/24 months	UV-X; IROC AG/ Riboflavin 0.1% w dextran 20%	Improved UDVA and CDVA. Reduced max-K and mean-K.	Mean max K (treated): 58.6 ± 9.62	Treated: -1.7 ± 3.9 (P < 0.001)	Treated: From 0.84 ± 0.34 to 0.77 ± 0.37 (LogMAR) (P = 0.04)	Treated: From 0.35 ± 0.24 to 0.23 ± 0.21 (LogMAR) (P < 0.001)	MRSE (treated): -0.86 (P = 0.07)
O’Brart *et al*, 2011 [2]	Blind, randomised, prospective, bilateral/Keratoconus	46; 24 treated, 22 FE control	18	1. ↓ UCVA/ BSCVA > 1 line 2. Worsening refractive/corneal astigmatism, K or cone apex power by 0.75D /18 months	In-house manufactured device using Roithner Lasertechnik diodes and CMB Vega X-linker/ Riboflavin 0.1%	Improved BSCVA. Reduced Orbscan II-simulated K, 3mm K, simulated astigmatism, cone apex power, root mean square, coma, spherical aberration, secondary astigmatism and pentafoil	Mean SIM K (treated): 47.1 Mean SIM K (control): 47.8	Treated: -0.62 (P < 0.001) Control: +0.14 (P = 0.3)	Treated: +0.06 (SDE) Control: -0.01 (SDE) (P = 0.2)	Treated: +0.12 (SDE) Control: +0.13 (SDE) (P = 0.01)	Mean SE (treated): +0.82 Mean SE (control): +0.11 (P = 0.2)
Wittig-Silva *et al*, 2008 [3]	Prospective, randomised/ Keratoconus	66: 33 treated; 33 control	12	1. ↑ ≥ 1D in Kmax 2. ↑ astigmatism with manifest subjective refraction ≥ 1D 3. ↑ of 0.50D in MRSE 4. ↓ ≥ 0.1mm in back optic zone radius of best fitting contact lens	IROC UV-X/ Riboflavin 0.1% w dextran 20%	Improved BSCVA and reduced Kmax.	Mean Kmax (treated): 52.70 ± 4.5 Mean Kmax (control): 50.80 ± 4.30 (P = 0.073)	Treated: -1.45 ± 1.00 (P < 0.002) Control: +1.28 (P < 0.001)	-	Treated: -0.12 (P = 0.07) (LogMAR)	MRSE: no diff in both treated and control
Wittig-Silva *et al*, 2014 [4]	Prospective, randomised/ Keratoconus	46 treated, 48 control	36	Subjective ↓ in vision and ≥ 1 of the following in 12 months: 1. ↑ ≥ 1D in steepest simulated K 2. ↑ astigmatism with manifest subjective refraction ≥ 1D 3. ↓ ≥ 0.1mm in back optic zone radius of best fitting contact lens	UV-X 1000; IROC/ Riboflavin 0.1% w dextran 20%	Improved UCVA and BSCVA, reduction in Kmax. Significant reduction in corneal thickness.	Kmax (treated): 52.87 ± 4.31 Kmax (control): 51.18 ± 4.03 (P = 0.052)	Treated: -1.03 ± 0.19 Control: +1.75 ± 0.38 (P < 0.001)	Treated: -0.15 ± 0.06 Control: +0.10 ± 0.04 (LogMAR) (P = 0.001)	Treated: -0.09 ± 0.03 Control: -0.05 ± 0.03 (LogMAR) (P = 0.347)	Treated: -0.61 ± 0.41 Control: -0.79 ± 0.42 (P = 0.752)
Lang *et al*, 2015 [30]	Prospective, randomised, blinded, placebo controlled/ Keratoconus	29	37	1. ↑ ≥ 1D in Kmax/1 year 2. Clinically significant Δ refraction	-/Riboflavin 0.1%	Corneal refractive power decreased in treatment group but increased in control group.	Kmax (treatment): 47.3 ± 2.2 Kmax (control): 50.9 ± 5.7 (P = 0.05)	Treatment: -0.35 ± 0.58 Control: +0.11 ± 0.61 (P = 0.02)	-	-	-

*UV* = Ultraviolet *Pre-op* = Pre-operative *FE* = Fellow-Eye *UCVA* = Uncorrected Visual Acuity *BSCVA* = Best Spectacle-Corrected Visual Acuity *BCVA* = Best Corrected Visual Acuity *UDVA* = Uncorrected Distance Visual Acuity *CDVA* = Corrected Distance Visual Acuity *Kmax* = maximum keratometry *max* = maximum *min* = minimum *K* = keratometry *D* = dioptre *cyl* = cylinder *SE* = spherical equivalent

*MRSE* = Manifest Refraction Spherical Equivalent *SDE* = Snellen Decimal Equivalent *w* = with *SIM* = Simulated

**Table 1B:** Summary of outcomes for standard epithelium-off cross-linking. (Prospective non-randomised studies) (3mW/cm^2^ UV-A exposure, 5.4J/cm^2^).

**Study**	**Study design/ Indication**	**No. of Eyes**	**Follow-up, months**	**Criteria for Progression**	**UV device/UV energy/ Riboflavin**	**Outcome**					
–	–	–	–	–	–	**Overall**	**Pre-op K (D)**	**ΔK (D)**	**ΔUCVA**	**ΔBCVA**	**Δ Refraction (D)**
Caporossi *et al*, 2006 [12]	Prospective non-randomised open/ Keratoconus	18; 10 treated, 8 FE control	6	-	Exerion-Sas/ Riboflavin 0.1% w dextran 20%	Reduction in mean spherical equivalent, improvement in morphologic symmetry with reduction in comatic aberrations.	-	**Δ** Mean K (treated): -2.1 ± 0.13 in central 3.0mm	Treated: 3.6 lines improvement (P = 0.0000112)	Treated: 1.66 lines improvement (Glasses) (P = 0.00071)	SE (treated): -2.5
Vinciguerra *et al*, 2009 [10]	Prospective, non-randomised single-center/ Keratoconus	56; 28 treated, 28 FE control	12	1. Δ myopia and/or cyl of ≥ 3D/6 months 2. Mean central Δ K ≥ 1.5D in 3 consecutive topographies/ 6 months 3. Mean CCT ↓ ≥ 5% in 3 consecutive tomographies/ 6 months	Peschke Meditrade/ Riboflavin 0.1% w dextran 20%	Improved UCVA and BSCVA, reduced APP and AK, reduced corneal and total wavefront aberrations.	SIM K steepest (treated): 50.37	Treated: -6.16 (P < 0.05)	Treated: From 0.17 ± 0.09 to 0.27 ± 0.08 (LogMAR) (P < 0.05)	Treated: From 0.52 ± 0.17 to 0.72 ± 0.16 (LogMAR) (P < 0.05)	SE (treated): From -3.36 ± 2.64 to -2.96 ± 2.68
Coskunseven *et al*, 2009 [17]	Prospective comparative/ Keratoconus	38; 19 treated, 19 FE control	5 - 12	Increase in maximum K by 1D / 6 months	Peschke Meditrade/ Riboflavin 0.1% w dextran 20%	Progression of keratoconus stopped. ↓ in corneal curvature, SE refraction, and refractive cylinder.	Kmax (treated): 54.02 ± 4.15 Kmax (control): 48.32 ± 3.00	Treated: -1.57 ± 1.14 (P < 0.01) Control: + 0.04 ± 1.34 (P = 0.446)	Treated: increased by 0.06 ± 0.05 (LogMAR) (P < 0.01) Control: decreased by 0.08 ± 0.12 (LogMAR) (P < 0.1)	Treated: increased by 0.1± 0.14 (LogMAR) (P < 0.01) Control: decreased by 0.06 ± 0.09 (LogMAR) (P < 0.01)	SE (treated): +1.03 ± 2.22 (P < 0.01) SE (control): + 0.03 ± 0.96 (P = 0.441)
Vinciguerra *et al*, 2009 [11]	Prospective, nonrandomized single-center/ Keratoconus	28 treated, 28 FE control	24	Documented keratoconus progression in the previous 6 months.	Peschke Meditrade/5.4J/cm^2^/Riboflavin 0.1% w dextran 20%	Improved UCVA and BSCVA, reduced APP and AK, reduced corneal and total wavefront aberrations.	Steepest SIM K: 50.37	SIM K: 50.37 to 49.02 (P = 0.03)	From 0.77 ± 0.18 to 0.53 ± 0.19 (LogMAR)	From 0.28 ± 0.09 to 0.13 ± 0.10 (LogMAR)	From -3.37 ± 2.64 to -2.56 ± 2.68 (P = 0.03)
Wollensak *et al*, 2003 [1]	Prospective, non-randomised/ Keratoconus	23	3 – 48 (Mean: 23.2 ±12.9)	Preoperative progression of K value: 1.42 ± 1.18 D in 6 months	370nm; Roithner Lasertechnik/ Riboflavin 0.1% w dextran 20%	Progression of keratoconus stopped, visual acuity improved slightly.	Max K: 48-72	-2.01 ± 1.74 (P = 0.0001)	NA	1.26 ± 1.5 (P = 0.026)	SE: -1.14 ± 2.18 (P = 0.030)
Agrawal, 2009 [15]	Retrospective nonrandomised open label/ Keratoconus	68	6 – 16 (mean follow-up: 10.05 ± 3.55)	1. ↑ max K of 1.00 D/1 year 2. Patient reports of deteriorating BCVA 3. Need for new contact lens fitting > once/2 years	CBM X Linker/Riboflavin 0.1%	BCVA improved slightly, astigmatism decreased, K value of the apex decreased, reduction in comatic aberrations	Mean max K: 53.26 ± 5.93	-2.47 (54%) (P = 0.004), stable (38%)	-	1 line improvement (54%), stable (28%) (P = 0.006)	Cyl: -1.2 ± 4.02
Arbelaez *et al*, 2009 [16]	Prospective, nonrandomized/ Keratoconus	20	12	1. ↑ maximum K readings in several measurements over 3-6 months 2. Changes in refraction 3. ↓ visual acuity and contact lens intolerance	UV-X device/ Riboflavin	Improved UCVA and BSCVA. Reduced average keratometry reading, manifest refraction sphere and manifest cyl	Kmax apex: 51.89 ± 7.99	-1.4 (P = 0.01)	4.15 line improvement (P = 0.002)	1.65 line improvement (P = 0.002)	Sphere: -1.26 (P=0.0033) Cyl: -1.25 (P = 0.0003)

*UV* = Ultraviolet *Pre-op* = pre-operative *FE* = fellow-eye *UCVA* = Uncorrected Visual Acuity *BSCVA* = Best Spectacle-Corrected Visual Acuity *BCVA* = Best Corrected Visual Acuity *APP* = Average Pupillary Power *AK* = Apical Keratometry *Kmax* = maximum keratometry *max* = maximum *K* = keratometry *D* = Dioptre *cyl* = cylinder *CCT* = Central Corneal Thickness *SE* = Spherical Equivalent *w* = with *SIM* = simulated

**Table 1C:** Summary of outcomes for standard epithelium-off cross-linking. (Case series) (3mW/cm^2^ UVA exposure, 5.4J/cm^2^).

**Study**	**Study design/ Indication**	**No. of Eyes**	**Follow-up, months**	**Criteria for Progression**	**UV device/ UV energy/ Riboflavin**	**Outcome**					
–	–	–	–	–	–	**Overall**	**Pre-op K (D)**	**ΔK (D)**	**ΔUCVA**	**ΔBCVA**	**Δ Refraction (D)**
Vinciguerra *et al*, 2012 [31]	Prospective, interventional case series/ Keratoconus	40	24	Documented keratoconus progression in the previous 3 months	CSO-VEGA X-linker/ Riboflavin 0.1% w dextran 20%	Improved UCVA and BSCVA. Reduced corneal asymmetry and total wavefront aberrations	SIM K Steepest: 51.48 ± 3.4	From 51.48 ± 3.4 to 50.21 ± 3.2 (P = 0.07)	From 0.79 ± 0.21 to 0.58 ± 0.18 (LogMAR) (P < 0.05)	From 0.39 ± 0.10 to 0.20 ± 0.09 (LogMAR) (P < 0.05)	Mean SE: -1.57 (P = 0.02)
Vinciguerra *et al*, 2010 [27]	Prospective case series/ Post-laser ectasia	13	12	1. Δ in myopia/ astigmatism of ≥ 3D/6 months 2. Mean Δ in central and/or pupillary K ≥ 1.50D in 3 consecutive topographies/6 months 3. Total mean CCT ↓ of ≥ 5% in 3 consecutive tomographies/6 months	Peschke Meditrade/ Riboflavin 0.1% w dextran 20%	Improved BSCVA. Reduced mean SE refraction and mean refractive sphere reduction.	SIM K steepest: 45.93 ± 6.03	From 45.93 ± 6.03 to 42.49 ± 4.88 (P > 0.05)	From 1.08 ± 0.43 to 0.94 ± 0.46 (LogMAR) (P > 0.05)	From 0.16 ± 0.14 to 0.06 ± 0.08 (LogMAR) (P < 0.05)	SE: From -4.16 ± 2.90 to -3.25 ± 2.05 (P > 0.05)
Salgado *et al*, 2011 [26]	Prospective case series/ Post-laser ectasia	22	12	Progressive keratectasia after refractive surgery	Peschke Meditrade/ Riboflavin 0.1% w dextran 20%	Improved BCVA, UCVA and max-K.	Max K: 44.12 ± 3.97	From 44.12 ± 3.97 to 44.43 ± 4.06 (P > 0.05)	From 0.53 ± 0.38 to 0.40 ± 0.27 (LogMAR) (P > 0.05)	From 0.19 ± 0.21 to 0.15 ± 0.14 (LogMAR) (P > 0.05)	SE: From -2.39 ± 2.03 to -2.07 ± 2.18 (LogMAR) (P > 0.05)
Ivarsen *et al*, 2013 [32]	Retrospective follow-up/ Keratoconus	28	22	1. ↑ max K ≥ 1.5D/3-6 months 2. ↓ vision and Δ refraction	IROC UV-X/ Riboflavin 0.1% w dextran 20%	Progression of keratoconus stopped, decreased max K.	Max K: 61.2 ± 3.7	From 61.2 ± 3.7 to 59.1 ± 3.7	-	Unchanged	-
Richoz *et al*, 2013 [29]	Retrospective, interventional case series/Post-laser ectasia	26	12-62 (mean follow-up: 25 ± 13)	↑ Kmax of anterior corneal surface, at 3.0mm from apex of ≥ 1D/12 months	Peschke Meditrade/ Riboflavin 0.1% w dextran 20%	Improved mean CDVA, reduced mean Kmax. Significantly reduced index of surface variance, index of vertical asymmetry, keratoconus index and central keratoconus index	Mean Kmax: 52.8 ± 5	-1.9 ± 1.9 (P < 0.001)	-	From 0.5 ± 0.3 to 0.2 ± 0.16 (LogMAR) (P < 0.001)	-

*UV* = Ultraviolet. *UCVA* = Uncorrected Visual Acuity. *BSCVA* = Best Spectacle-Corrected Visual Acuity. *BCVA* = Best Corrected Visual Acuity.

*CDVA* = Corrected Distance Visual Acuity. *Kmax* = maximum keratometry. *max* = maximum. *K* = Keratometry. *D* = Dioptre.

*CCT* = Central Corneal Thickness. *SE* = Spherical Equivalent. *pre-op* = pre-operative. *w* = with *SIM* = simulated.

**Table 2:** Summary of outcomes for long-term studies of standard epithelium-off cross-linking.

**Study**	**Study design/ Indications**	**No. of Eyes**	**Follow-up, months**	**Criteria for Progression**	**UV device/ Riboflavin**	**Outcome**					
–	–	–	–	–	–	**Overall**	**Pre-op K (D)**	**Δ K (D)**	**Δ UCVA**	**Δ BCVA**	**Δ Refraction (D)**
Caporossi *et al*, 2010 [21]	Prospective, nonrandomised, open long-term trial/ Keratoconus	88; 44 treated, 44 FE control	48	-	CSO Vega CBM X linker/ Riboflavin 0.1% w dextran 20%	Reduced mean K value, reduced coma aberration. Improved BSCVA and UCVA.	-	Mean K (treated): -2.26 ± 0.68 Mean K (FE control): +2.2 ± 1.24	Treated: +2.85 ± 0.81 (Snellen lines)	Treated: +2.03 ± 1.04 (Snellen lines)	SE (treated): +2.15 ± 1.19 (P = 5.1 x 10^-10^
O’Brart *et al*, 2015 [24]	Prospective cohort study/ Keratoconus	65; 36 treated, 29 FE control	84	1. ↓ UDVA/ CDVA by > 1 line 2. deteriorating refractive/ corneal astigmatism, SIM K/ Kmax by 0.75D/12-24 months	-/ Riboflavin 0.1% w dextran 20%	Improvements in topographic and wavefront parameters evident at 1 year continue to improve after 7 years.	Mean Kmax (treated): 48.23 ± 3.49 Mean Kmax (FE control): 47.01 ± 3.54	Treated: -0.91 (P < 0.001) FE control: +0.86 (P < 0.05)	Treated: From 0.32 ± 0.26 to 0.46 ± 0.5 (SDE) (P < 0.0005) FE control: From 0.56 ± 0.4 to 0.43 ± 0.37 (SDE) (P = 0.4)	Treated: From 0.85 ± 0.25 to 0.96 ± 0.17 (SDE) (P < 0.0001) FE control: 0.91 ± 0.28 to 0.92 ± 0.29 (SDE) (P = 0.9)	Mean SE (treated): +0.78 (P <0.005) Mean SE (FE control): -1.66 ± 2.51 to -1.72 ± 2.27 (P = 0.8)
O’Brart *et al*, 2013 [19]	Follow-up study/ Keratoconus	30	48-72	1. ↓ UDVA/ CDVA by > 1 line 2. ↓ refractive/ corneal astigmatism, K or cone apex power by 0.75D/12-24 months	-/Riboflavin 0.1% w dextran 20%	Reduced mean spherical equivalent, mean simulated K, cone apex power. Improved CDVA.	Mean SIM K: 46.44 ± 3.4	From 46.44 ± 3.4 to 45.6 ± 3.3 (P < 0.001)	From +0.27 ± 0.29 to +0.286 ± 0.31 (SDE) (P = 0.6)	From 0.8 ± 0.27 to 0.905 ± 0.2 (SDE) (P < 0.04)	SE: From -1.61 ± 1.97 to -0.79 ± 1.7 (P < 0.001)
Hashemi *et al*, 2013 [20]	Prospective case series/ Keratoconus	40	60	1. ↑ ≥ 1D in max K, manifest cyl error or MRSE 2. ↓ ≥ 2 lines of BCVA	UV-X IROC/ Riboflavin 0.1% w dextran 20%	Improved BCVA. No change in mean K and max K, UCVA, and astigmatism.	Max K: 49.37 ± 3.48	From 49.37 ± 3.48 to 49.13 ± 3.29 (P = 0.645)	From 0.67 ± 0.52 to 0.65 ± 0.51 (LogMAR) (P = 0.853)	From 0.31 ± 0.28 to 0.19 ± 0.20 (LogMAR) (P = 0.016)	Mean MRSE: From -3.18 ± 2.23 to -2.77 ± 2.18 (P = 0.174)
Ucakhan *et al*, 2016 [22]	Prospective follow-up study/ Keratoconus	40	48	↑ > 1D in Kmax/12 months	UV-X, IROC/ Riboflavin 0.1% w dextran 20%	Improved UCVA and BSCVA. Reduced mean Kmax.	Mean Kmax: 58.4 ± 5.5	-1.2 ± 2.2 (P = 0.0046)	- 0.4 ± 0.2 (LogMAR) (P = 0.0001)	- 0.2 ± 0.2 (LogMAR) (P = 0.0001)	MRSE: From -6.2 ± 3.5 to -5.4 ± 3.8 (P = 0.04)
Raiskup-Wolf *et al*, 2008 [18]	Long-term retrospective study/ Keratoconus	241	Max 72	1. ↑ max K 1D/1 year 2. ↓ visual acuity 3. New CL/2 years	Fa. Peschke/ Riboflavin 0.1%	Reduction in steepening, improved BCVA	Kmax: 53.7 ± 7.5	-2.57 ± 3.71	-	-0.15 ± 0.18	-
Raiskup *et al*, 2015 [23]	Retrospective interventional case series/ Keratoconus	34	132 (Mean: 131.9 ± 20.1)	↑ apical K ≥ 1D/6-12 months	-	Reduced AK value, max K and min K. Improved CDVA. ECC is unchanged.	-	-	-	-0.14 (LogMAR) (P = 0.002)	-

*UV* = Ultraviolet *pre-op* = pre-operative UCVA = Uncorrected Visual Acuity *BSCVA* = Best Spectacle-Corrected Visual Acuity

*BCVA* = Best Corrected Visual Acuity UDVA = Uncorrected Distance Visual Acuity *CDVA* = Corrected Distance Visual Acuity *AK* = Apical Keratometry *Kmax* = maximum keratometry *max* = maximum *min* = minimum *K* = keratometry *ECC* = Endothelial Cell Count *SDE* = Snellen Decimal Equivalent

*D* = diopters *FE* = fellow eye *w* = with *SE* = spherical equivalent *SIM* = simulated *cyl* = cylinder

*MRSE* = Manifest Refraction Spherical Equivalent *CL* = Contact Lens

**Table 3A:** Summary of outcomes for epithelium-on (transepithelial) cross-linking (Adults).

**Study**	**Study design/ Protocol**	**No. of Eyes**	**Follow-up, months**	**Criteria for Progression**	**UV device/ UV energy/ Riboflavin**	**Outcome**					
–	–	–	–	–	–	**Overall**	**Pre-op K (D)**	**ΔK (D)**	**ΔUCVA**	**ΔBCVA**	**Δ Refraction (D)**
Soeters *et al*, 2015 [42]	Randomised clinical trial/ 3mW/cm^2^ 30 min	61; 35 epi-on, 26 epi-off	12	↑ Kmax, Ksteep, mean K and/or topographic cyl value by ≥ 0.5D/6-12 months	For both: UV-X; Peschke Meditrade Epi-on: 0.1% riboflavin with 15.0% dextran, trometamol and EDTA Epi-off: isotonic riboflavin 0.1% solution with 20% dextran	Average Kmax remained stable for the epi-off group but showed significant flattening in the epi-off group. CDVA showed a better outcome in the epi-on group.	Kmax (epi-off): 57.8 ± 7.1 Kmax (epi-on): 56.4 ± 5.0	Epi-off: -1.5 ± 2.0 Epi-on: +0.3 ± 1.8 (P = 0.022)	Epi-off: -0.15 ± 0.43 (LogMAR) Epi-on: -0.06 ± 0.37 (LogMAR) (P = 0.591)	Epi-off: -0.07 ± 0.21 (LogMAR) Epi-on: -0.14 ± 0.21 (LogMAR) (P = 0.023)	SE (Epi-off): +0.4 ± 3.0 SE (Epi-on): +0.3 ± 1.6 (P = 0.436)
Al Fayez *et al*, 2015 [41]	Prospective clinical trial/ 3mW/cm^2^ 30 min	70; 34 epi-on, 36 epi-off	36	↑ max K/ manifest astigmatism ≥ 1D/12 months	Epi-on: IROC/ 1% tetracaine/ 0.02% benzalkonium chloride, dextran-free riboflavin Epi-off: IROC/ 0.1% riboflavin with dextran 20% solution 30 min	Kmax decreased in the epi-off group but increased in epi-on group.	-	Kmax (epi-off): -2.4 Kmax (epi-on): +1.1 (P < 0.0001)	Epi-off: -0.2 (LogMAR) Epi-on: +0.1 (LogMAR) (P < 0.0001)	Epi-off: -0.1 (LogMAR) Epi-on: +0.06 (LogMAR) (P = 0.055)	-
Filippello *et al*, 2012 [37]	Prospective case-control cohort study/ 3mW/cm^2^ 30 min	40; 20 epi-on, 20 FE control	18	1. ↑ max cone apex curvature ≥ 1D/6 months 2. ↓ corneal thickness > 2%/6 months 3. ↑ central corneal astigmatism ≥ 1D/6 months	Vega/ 0.1% riboflavin with dextrane T500, trometamol and EDTA sodium salt	Improved UCVA and CVA, topography-derived keratometry, cone apex power, and HOA.	SIM K steepest (treated): 51.02 ± 1.10 SIM K steepest (FE control): 51.12 ± 1.02	Treated: From 51.02 ± 1.10 to 48.05 ± 0.21 FE control: 51.12 ± 1.02 to 52.12 ± 0.47 (P < 0.05)	Treated: From 0.71 ± 0.12 to 0.48 ± 0.34 (LogMAR) FE control: From 0.84 ± 0.23 to 0.98 ± 0.41 (LogMAR) (P < 0.05)	Treated: From 0.35 ± 0.23 to 0.24 ± 0.77 (LogMAR) FE control: From 0.46 ± 0.21 to 0.64 ± 0.39 (LogMAR) (P < 0.05)	-
Leccisotti *et al*, 2010 [47]	Prospective, consecutive, single-masked, paired-eye study/ 3mW/cm^2^ 30 min	102; 51 treated, 51 FE control	12	Myopia/ astigmatism ↑ 1D or average SIM K ↑ 1.50D/12 months	CBM Vega X-linker/ 0.1% riboflavin with 20% dextran T500 and oxybuprocaine	Improved mean CDVA, decreased mean SE refraction, reduced increase of mean apex curvature, decreased mean average simulated K, reduced increase of mean index of surface variance.	Mean average SIM K (treated): 46.63 ± 2.89 Mean average SIM K (control): 44.60 ± 2.19	Treated: -0.10 ± 1.44 Control: 0.88 ± 2.35 (P < 0.05)	-	Treated: -0.036 ± 0.049 (LogMAR) Control: +0.039 ± 0.032 (LogMAR) (P < 0.05)	Mean SE (treated): +0.35 ± 0.66 Mean SE (control): -0.83 ± 0.88 (P < 0.05)
Vinciguerra *et al*, 2014 [44]	Prospective non-randomised clinical study/ 10mW/cm^2^ 9 min	20	12	1. Δ curvature in cone area of ≥ 1D 2. Thinning of > 20μm in minimal Scheimpflug corneal thickness	UV-X 2000; IROC/ 0.1% riboflavin, with EDTA and trometamol, dextran-free or sodium chloride administered by iontophoresis (I-ON XL, SOOFT)	Improved CDVA. Aberrometry remained stable and a trend towards improvement. No progression of keratoconus.	Max K: 59.07 ± 3.90	-0.549 ± 2.344 (P = 0.40)	-	-0.12 ± 0.06 (LogMAR) (P = 0.01)	SE: +1.117 ± 3.783 (P = 0.20)
Koppen *et al*, 2012 [48]	Prospective cohort study/ 3mW/cm^2^ 30 min	53	18	1. ↑ max K ≥ 1D 2. ↓ visual acuity and refraction	Vega CBM X-linker/ 0.1% riboflavin in 20.0% dextran	Only corrected distance visual acuity showed significant improvement. Maximum K and pachymetry at the thinnest point continued to progress.	SIM K steepest: 48.69 ± 5.39	+0.48 ± 0.28 (P > 0.05)	-	+0.05 ± 0.03 (SDE) (P > 0.05)	Sphere: + 0.04 ± 0.21 (P > 0.05) Cyl: -0.08 ± 0.19 (P > 0.05)
Caporossi *et al*, 2013 [40]	Prospective case series/ 3mW/cm^2^ 30 min	26	24	1. ↓ UDVA and/or CDVA > 1 Snellen line 2. ↑ sphere and/or cyl > 0.50 D 3. ↑ topographic symmetry index surface asymmetry index and/or symmetry index > 0.50D 4. ↑ max K > 1D 5. ↓ thinnest point on AC OCT ≥ 10μm	CBM X-linker, VEGA/ 5.4J/cm^2^/ 0.1% riboflavin with 15.0% dextran, trometamol and EDTA	UDVA and CDVA improved in the first 3-6 months but returned to baseline. Simulated maximum K value worsened at 24 months. Spherical aberration increased at 24 months.	Max K: 48.59	+1.55 (P = 0.05)	-0.05 Snellen lines (P = 0.61)	+0.05 Snellen lines (P = 0.57)	-
Bikbova *et al*, 2014 [43]	Prospective case series/ 3mW/cm^2^ 30 min	22	12	1. ↑ steepest K by ≥ 1D in manifest cyl 2. ↑ ≥ 0.5D in manifest SE	UFalink/ Riboflavin 0.1% solution administered by iontophoresis (Potok-1)	Decreased average K level, corneal astigmatism. Improved UDVA.	Max K: 47.82 ± 2.23	From 47.82 ± 2.23 to 45.72 ± 2.13	From 0.61 ± 0.44 to 0.48 ± 0.41	From 0.34 ± 0.29 to 0.29 ± 0.25 (LogMAR) (P > 0.062)	Cyl: From 3.44 ± 0.48 to 2.95 ± 0.23

*UV* = Ultraviolet *pre-op* = pre-operative *FE* = Fellow-Eye *UCVA* = Uncorrected Visual Acuity *BCVA* = Best Corrected Visual Acuity

*UDVA* = Uncorrected Distance Visual Acuity *CDVA* = Corrected Distance Visual Acuity *CVA* = Corrected Visual Acuity *Kmax* = maximum keratometry

*Ksteep* = steepest keratometry *K* = keratometry *epi-on* = epithelium-on *epi-off* = epithelium-off *EDTA* = sodium ethylenediaminetetraacetic acid *Trometamol* = Tris-hydroxymethyl aminomethane *SE* = Spherical Equivalent *HOA* = Higher-Order Abberations

*AC OCT* = Anterior Chamber Optical Coherence Tomography *D* = Diopters *cyl* = cylinder *max* = maximum *SIM* = simulated

**Table 3B:** Summary of outcomes for epithelium-on (transepithelial) cross-linking (Pediatrics).

**Study**	**Study design/ Protocol**	**No. of eyes**	**Follow-up, months**	**Criteria for Progression**	**UV device/ UV energy/ Riboflavin**	**Outcome**					
						**Overall**	**Pre-op K (D)**	**ΔK (D)**	**ΔUCVA**	**ΔBCVA**	**Δ Refraction (D)**
Salman, 2013 [38]	Prospective comparative case series/ 3mW/cm^2^ 30 min	44; 22 epi-on, 22 FE control	12	1. K > 45.0D 2. Inferior steepening > 1.0D in superior half of cornea 3. 1.0D of tomographic cyl progression/1 year 4. ↓ CDVA 5. New CL fitting/2 years	Opto XLink/ 5.4J/cm^2^/ 0.1% riboflavin with 15.0% dextran, trometamol and EDTA	Improved mean UDVA, decreased mean simulated K, mean flattening of apical K	Mean SIM K (treated): 49.98 ± 4.46 Mean SIM K (FE control): 48.78 ± 3.46	Mean SIM K (treated): -2.03 (P < 0.05) Mean SIM K (FE control): +0.59 (P > 0.05)	Treated: From 0.95 ± 0.34 to 0.68 ± 0.45 (LogMAR) (P < 0.023) FE control: From 0.84 ± 0.52 to 0.94 ± 0.22 (LogMAR) (P = 0.324)	Treated: From 0.51 ± 0.11 to 0.49 ± 0.09 (LogMAR) (P = 0.189) FE control: From 0.42 ± 0.11 to 0.51 ± 0.21 (LogMAR) (P = 0.543)	SE (treated): -From 3.17 ± 2.72 to -2.87 ± 2.86 (P = 0.751) SE (control): -3.72 ± 4.72 to -4.12 ± 2.42 (P = 0.032)
Buzzonetti *et al*, 2012 [36]	Prospective case series/ 3mW/cm^2^ 30 min	13	18	-	CBM X-linker, VEGA/ 0.1% riboflavin with 15.0% dextran, trometamol and EDTA	Improved CDVA but K readings and HOAs showed significant worsening	Kmax: 48.90 ± 3.60	From 48.90 ± 3.60 to 52.90 ± 4.90 (P < 0.05)	-	From 0.19 ± 0.14 to 0.1 ± 0.1 (LogMAR) (P < 0.05)	SE: From -3.10 ± 2.40 to -3.50 ± 2.90
Buzzonetti *et al*, 2015 [45]	Prospective case series/ 10mW/cm^2^ 9 min	14	15	-	-/Riboflavin solution administered by iontophoresis (I-ON CXL)	CDVA improved from 0.7 ± 1.7 to 0.8 ± 1.8. Unchanged SE, refractive astigmatism, topographic and aberrometric data. Unchanged mean thinnest point and endothelial cell density.	Kmax: 47.6 ± 2.0	From 47.6 ± 2.0 to 48.0 ± 2.3 (P = 0.08)	-	From 0.7 ± 1.7 to 0.8 ± 1.8 (LogMAR) (P = 0.005)	From -2.2 ± 2.7 to -1.5 ± 1.8 (P = 0.3)
Magli *et al*, 2016 [46]	Prospective case series/ 10mW/cm^2^ 9 min	13	18	↑ max cone apex curvature ≥ 1D/6 months	UV-X 2000; IROC/ Riboflavin 0.1% with EDTA and tromethamine without dextran or sodium chloride administered by iontophoresis (I-ON XL, SOOFT)	Stabilisation of refractive UCVA and BCVA as early as 1 month after CXL. Kmax remained stable. Pediatric keratoconus progression halted.	Kmax: 53.26 ± 3.88	From 53.26 ± 3.88 to 53.98 ± 7.94 (P = 0.04)	From 0.67 ± 0.22 to 0.63 ± 0.36 (LogMAR) (P = 0.05)	From 0.45 ± 0.28 to 0.42 ± 0.22 (LogMAR) (P = 0.03)	-
Magli *et al*, 2013 [39]	Retrospective/ 3mW/cm^2^ 30 min	37; 14 epi-on, 23 epi-off	12	↑ max cone apex curvature ≥ 1D/6 months	Epi-on: Vega/ 0.1% riboflavin with 15.0% dextran, trometamol and EDTA Epi-off: Vega CBM X linker/ 0.1% riboflavin in 20% dextran	Significant reduction in Kmax, Kmin, mean K in both the epi-off and epi-on groups.	Epi-off: 50.13 ± 4.0 Epi-on: 49.27 ± 4.1	Kmax (Epi-off): -1.11 (P = 0.01) Kmax (Epi-on): -1.14 (P = 0.02)	Epi-off: From 0.68 ± 0.21 to 0.67 ± 0.24 (LogMAR) (P = 0.1) Epi-on: From 0.55 ± 0.33 to 0.54 ± 0.22 (LogMAR) (P = 0.3)	Epi-off: From 0.36 ± 0.1 to 0.36 ± 0.1 (LogMAR) (P = 0.8) Epi-on: From 0.26 ± 0.2 to 0.27 ± 0.2 (LogMAR) (P = 0.5)	-

*UV* = Ultraviolet *pre-op* = pre-operative *FE* = Fellow-Eye *UCVA* = Uncorrected Visual Acuity *BCVA* = Best Corrected Visual Acuity

*UDVA* = Uncorrected Distance Visual Acuity *CDVA* = Corrected Distance Visual acuity *Kmax* = maximum keratometry *Kmin* = minimum keratometry

*K* = keratometry *epi-on* = epithelium-on *epi-off* = epithelium-off *EDTA* = sodium Ethylenediaminetetraacetic Acid

*Trometamol* = Tris-hydroxymethyl aminomethane *SE* = Spherical Equivalent *CXL* = Cross-Linking *HOA* = Higher-Order Abberations *CL* = Contact Lens *D* = diopters *cyl* = cylinder *SIM* = simulated *max* = maximum

**Table 4A:** Summary of outcomes for accelerated cross-linking (comparative studies).

**Study**	**Study design/ Indication/ Protocol**	**No. of Eyes**	**Follow-up, months**	**Criteria for Progression**	**UV device/ Riboflavin**	**Protocol**	**Outcome**					
–	–	–	–	–	–	–	**Overall**	**Pre-op K (D)**	**ΔK (D)**	**Δ UCVA**	**Δ BCVA**	**Δ Refraction (D)**
Kanellopoulos, 2012 [51]	Prospective, randomised bilateral comparison trial/ Keratoconus	42; 21 Group A (treated), 21 Group B (FE control)	18-56 (mean 46)	K > 45 and/or inferior steepening > 1D to the superior half of the cornea and 1D of tomographic cyl progression/ 1 year	-/ 0.1% riboflavin 5 min	Group A: 7mW/cm^2^ 15 min Group B: 3mW/cm^2^ 30 min	Improved UDVA and BCVA in both groups. Reduced mean sphere, mean cyl and steepest K.	-	Group A: 49.5 to 46.1 Group B: From 48.7 to 45.8	Group A: From 20/60 to 20/38 Group B: From 20/62 to 20/40	Both groups: From 20/30 to 20/25	SE (Group A): -2.5 SE (Group B): -2.3
Shetty *et al*, 2015 [56]	Prospective randomised interventional study/ Keratoconus	138; 36 Group 1, 36 Group 2, 33 Group 3, 33 Group 4	12	↑ steep K by > 1.0-1.5D, a corresponding Δ (>1.0-1.5D) in subjective refraction or a ↓ ≥ 5% in thinnest pachymetry/ 6 months	Avedro KXL/ 0.1% riboflavin with 20% dextran 30 min	Group 1: 3mW/cm^2^ 30 min Group 2: 9mW/cm^2^ 10 min Group 3: 18mW/cm^2^ 5 min Group 4: 30mW/cm^2^ 3 min	Improved mean CDVA and SE in all groups except Group 4, with Group 3 showing the best results. Flattening of steep and flat K was significant in Groups 1 and 2. Groups 1 and 2 showed a good demarcation line.	Steep K (Group 1): 50.5 ± 4.2 Steep K (Group 2): 49.9 ± 3.8 Steep K (Group 3): 48.6 ± 3.5 Steep K (Group 4): 49.4 ± 4.2 (P = 0.23)	Group 1: 1.32 (P < 0.001) Group 2: 0.67 (P < 0.006) Group 3: 0.52 (P < 0.03) Group 4: -0.18	-	Group 1: 0.04 (SDE) (P < 0.05) Group 2: 0.06 (SDE) (P < 0.05) Group 3: 0.10 (SDE) (P < 0.05) Group 4: 0.02 (SDE) (P < 0.05)	Group 1: -0.85 (P < 0.01) Group 2: -1 (P < 0.01) Group 3: -1.68 (P < 0.01) Group 4: -0.49 (P = 0.12)
Sherif, 2014 [58]	Prospective randomised interventional case-control clinical trial/ Keratoconus	25; 14 accelerated, 11 conventional	12	↑ ≥ 1.0D in steepest K, ↑ ≥ 1.0D in manifest cyl, or ↑ ≥ 0.5D in MRSE/ 6 months	0.1% riboflavin with dextran 30 min	Accelerated: 30mW/cm^2^ 4min 20s Conventional: 3mW/cm^2^ 30 min	Decreased flat K, steep K and mean K in both groups. Improved BSCVA.	Max K (accelerated): 49.43 ± 1.63 Max K (conventional): 51.4 ± 1.69	Accelerated: From 49.43 ± 1.63 to 48.2 ± 1.43 (P = 0.022) Conventional: From 51.4 ± 1.69 to 50.24 ± 2 (P = 0.099)	-	Accelerated: From 0.48 ± 0.17 to 0.61 ± 0.15 (SDE) (P=0.015) Conventional: From 0.49 ± 0.19 to 0.64 ± 0.16 (SDE) (P = 0.03)	-
Ng *et al*, 2016 [59]	Comparative interventional study/ Keratoconus	26; 12 accelerated, 14 conventional	14	↑ >1D in Kmax, ↑ >1D in manifest cyl or ↑ >0.5D in SE over 6-12 months	Conventional: UV-X 1000, IROC/ Accelerated: UV-X 2000, IROC For both: Isotonic 0.1% riboflavin with 20% dextran solution 25 min	Accelerated: 9mW/cm^2^ 10 min Conventional: 3mW/cm^2^ 30 min	Conventional: improved CDVA, reduced Kmax, Kmean. Accelerated: unchanged CDVA, Kmax, Kmean	Kmax (conventional): 53.5 ± 6.3 Kmax (accelerated): 51.6 ± 4.0 (P = 0.820)	Conventional: -1.8 ± 1.8 Accelerated: -0.3 ± 0.9 (P = 0.015)	-	Conventional: -0.126 ± 0.194 (LogMAR) Accelerated: 0.021 ± 0.092 (LogMAR) (P = 0.060)	SE (conventional): 0.23 ± 0.87 SE (accelerated): 0.98 ± 3.81 (P = 0.796)
Chow *et al*, 2015 [60]	Prospective, interventional clinical study/ Keratoconus	38; 19 accelerated, 19 conventional	12	↓ ≥ 2 lines of BCVA + ≥ 1 of the following/ 12 months: 1. ↑ ≥ 1D in steepest K 2. ↑ ≥ 1D in astigmatism	Conventional: UV-X, IROC Accelerated: CCL-Vario, Peschke Trade GmbH For both: 0.1% riboflavin with 20% dextran solution 30 min	Accelerated: 18mW/cm^2^ 5 min Conventional: 3mW/cm^2^ 30 min	Improved UCVA and BCVA, reduction in SE in both groups. A more effective topographic flattening was observed in conventional CXL.	Max K (conventional): 54.93 ± 1.72 Max K (accelerated): 51.96 ± 1.80 (P = 0.235)	Conventional: -1.6 ± 0.72 Accelerated: -0.47 ± 0.83 (P = 0.343)	Conventional: -0.28 ± 0.08 (LogMAR) Accelerated: -0.20 ± 0.06 (LogMAR) (P = 0.508)	Conventional: 0.00 ± 0.04 (LogMAR) Accelerated: -0.14 ± 0.02 (LogMAR) (P = 0.430)	SE (conventional): -1.3 ± 0.53 SE (accelerated): -0.57 ± 0.26 (P = 0.554)
Hashemian *et al*, 2014 [61]	Prospective clinical trial/ Keratoconus	153; 77 accelerated, 76 conventional	15	Δ Mean central K ≥ 1.5D and ↓ > 5% in mean CCT through 3 consecutive readings/ 6 months	CCL-VARIO, Peschke Meditrade GmbH/ 0.1% riboflavin with 20% dextran solution 30 min	Accelerated: 30mW/cm^2^ 3 min Conventional: 3mW/cm^2^ 30 min	Cyl and spherical components of refraction improved significantly. No difference observed between the 2 groups.	-	Kmax (conventional): -1.98 ± 0.93 Accelerated: -1.85 ± 0.99 (P = 0.36)	Conventional: 0.21 ± 0.19 (LogMAR) Accelerated: 0.19 ± 0.20 (LogMAR) (P = 0.64)	Conventional: 0.17 ± 0.10 (LogMAR) Accelerated: 0.16 ± 0.09 (LogMAR) (P = 0.58)	Sphere (conventional): From -4.3 ± 1.6 to -2.9 ± 2.0 Sphere (accelerated): -4.8 ± 1.9 to -3.5 ± 2
Tomita *et al*, 2014 [53]	Prospective comparative study/ Keratoconus	48; 30 accelerated, 18 conventional	12	-	Accelerated: Avedro KXL/ 0.1% riboflavin with HPMC 15 min Conventional: CCL-365 Vario, Peschke Meditrade/ 0.1% riboflavin with 20.0% dextran T500 30 min	Accelerated: 30mW/cm^2^ 3 min Conventional: 3mW/cm^2^ 30 min	Both accelerated and conventional CXL were safe and effective. Similar morphologic changes and a pronounced demarcation line were apparent in eyes in both groups postoperatively.	Mean Kmax (accelerated): 50.45 ± 5.28 Mean Kmax (conventional): 48.82 ± 4.56	Accelerated: -0.62 ± 1.46 Conventional: -1.77 ± 2.65 (P = 0.21)	-	-	MRSE (accelerated): 0.64 ± 1.84 MRSE (conventional): 0.39 ± 0.88 (P = 0.60)
Kymionis *et al*, 2014 [54]	Prospective comparative interventional case series/ Keratoconus	21; 12 accelerated, 9 conventional	1	-	CCL-365, Peschke Meditrade/ 0.1% riboflavin with 20% dextran 30 min	Accelerated: 9mW/cm^2^ 10 min Conventional: 3mW/cm^2^ 30 min	The mean corneal stroma demarcation line depth was 350.78 mum ± 49.34 in the conventional group and 288.46 ± 42.37 mum in the accelerated group.	Mean K steep (conventional): 49.35 ± 2.80 Mean K steep (accelerated): 47.58 ± 2.83 (P = 0.17)	-	-	-	-
Kymionis *et al*, 2014 [55]	Prospective comparative study/ Keratoconus	52; 26 accelerated, 26 conventional	1	-	CCL-365, Peschke Meditrade/ 0.1% riboflavin with 20% dextran 30 min	Accelerated: 9mW/cm^2^ 14 min Conventional: 3mW/cm^2^ 30 min	Corneal stromal demarcation line depth showed no significant difference for both groups.	Mean steep K (conventional): 49.88 ± 3.99 Mean steep K (accelerated): 49.17 ± 2.90 (P = 0.467)	-	-	-	-
Mazzotta *et al*, 2014 [57]	Prospective, comparative, interventional clinical study/ Keratoconus	20; 10 accelerated pulsed, 10 accelerated continuous	12	↓ UCVA/ BSCVA > 0.50 Snellen lines, ↑ sphere/cyl > 0.50D, ↑ topographic symmetry index SAI/SI > 1D, ↑ mean K > 1D or ↓ thinnest point at corneal OCT pachymetry ≥ 10μm	Avedro KXL/ 0.1% riboflavin dextran-free 10 min	Pulsed: 30mW/cm^2^ 8 min Continuous: 30mW/cm^2^ 4 min	Better functional outcomes and deeper stromal penetration in pulsed light accelerated treatment.	-	Apical K (Continuous): -1.39 ± 0.38 (P = 0.05) Apical K (Pulsed): +0.15 ± 0.8 (P = 0.077)	Pulsed: +0.9 ± 1.1 (SDE) (P = 0.10) Continuous: +0.5 ± 1.2 (SDE) (P = 0.65)	Pulsed: +1.8 ± 1.3 (SDE) (P = 0.55) Continuous: +1.6 ± 1.0 (SDE) (P = 0.56)	-
Woo *et al*, 2017 [62]	Prospective, non-randomised interventional study	76; 47 accelerated, 29 conventional	12	1. ↑ ≥ 1D in steepest K 2. ↓ > 5% in minimal corneal thickness 3. ↑ >1D in cyl/ >0.50D SE over ≥ 6 months	Conventional: UV-X, Peschke Meditrade/ isotonic riboflavin 0.1% with dextran 20% 30 min Accelerated: Avedro KXL/ dextran-free riboflavin 0.1% 10 min	Conventional: 3mW/cm^2^ 30 min Accelerated: 30mW/cm^2^ 4 min	Both groups showed no significant increase in K1, K2 and Kmean from baseline at 12 months. No difference between CXL and KXL group for postoperative corneal topography and central and minimal pachymetry/ 12 months.	Steepest K (conventional): 52.29 ± 5.40 Steepest K (accelerated): 52.15 ± 5.30 (P = 0.915)	Conventional: -0.13 Accelerated: -0.21 (P = 0.829)	Conventional: -0.11 (LogMAR) (P = 0.017) Accelerated: no statistically significant change	Conventional: -0.11 (LogMAR) (P = 0.037) Accelerated: -0.08 (P = 0.004)	SE (conventional): From -4.72 ± 3.6 to -3.82 ± 4.4 (P = 0.247) SE (accelerated): From -4.30 ± 3.1 to -5.11 ± 4.07 (P = 0.131)

*UV* = ultraviolet *pre-op* = pre-operative *FE* = Fellow-Eye *UCVA* = Uncorrected Visual Acuity *BSCVA* = Best Spectacle-Corrected Visual Acuity *BCVA* = Best Corrected Visual Acuity *UDVA* = Uncorrected Distance Visual Acuity *CDVA* = Corrected Distance Visual Acuity *Kmax* = maximum keratometry *Kmean* = mean keratometry *K* = keratometry *HPMC* = Hydroxypropyl Methylcellulose *SE* = Spherical Equivalent *CXL* = Cross-Linking *KXL* = Accelerated cross-linking *CCT* = Central Corneal Thickness

*cyl* = cylinder *OCT* = Optical Coherence Tomography *SAI* = Surface Asymmetry Index *SI* = Symmetry Index *D* = Diopters *SDE*: Snellen Decimal Equivalent

*MRSE* = Manifest Refractive Spherical Equivalent *max* = maximum

**Table 4B:** Summary of outcomes for accelerated cross-linking (non-comparative studies).

**Study**	**Study design/ Indication/ Protocol**	**No. of eyes**	**Follow-up, months**	**Criteria for Progression**	**UV device/ Riboflavin**	**Protocol**	**Outcome**					
							**Overall**	**Pre-op K (D)**	**ΔK (D)**	**Δ UCVA**	**Δ BCVA**	**Δ Refraction (D)**
Gatzioufas *et al*, 2013 [52]	Prospective cohort study/ Keratoconus	7	6	Mean of 3 consecutive measurements showing ↑ Kmax > 1D/ 12 months	CXL-365 Vario/ 0.1% riboflavin with 20% dextran 30 min	18mW/cm^2^ 5 min	Kmax, Kmean and CDVA showed no significant changes after 6 months. No complications were observed postoperatively.	Kmax: 55.6 ± 3.8	From 55.6 ± 3.8 to 52.9 ± 2.7 (P = 0.42)	-	From 0.41 ± 0.34 to 0.58 ± 0.37 (LogMAR) (P = 0.055)	-
Shetty *et al*, 2014 (paeds) [63]	Prospective case series/ Keratoconus	30	24	↑ steep K >1.0-1.5D and Δ in subjective refraction/ 6 months or ↓ ≥5% in thinnest pachymetry/ 6 months	Avedro KXL/ 0.1% riboflavin with 20% dextran for 30 min	9mW/cm^2^ for 10 min	Improved mean UDVA, mean CDVA, mean spherical refraction, mean cyl, and SE	Max K: 53.77 ± 4.82	From 53.77 ± 4.82 to 51.70 ± 5.41 (P = 0.007)	From 0.76 ± 0.26 to 0.61 ± 0.25 (LogMAR) (P = 0.005)	From 0.24 ± 0.19 to 0.12 ± 0.12 (LogMAR) (P < 0.001)	Mean SE: from -4.70 ± 3.86 to -3.75 ± 3.49 (P = 0.15)
Marino *et al*, 2015 [64]	Prospective, single-center case series/ Post-laser ectasia	40	24	1. ↑ inferior steepening 2. ↑ myopia and astigmatism 3. ↓ UDVA and CDVA	CCL-Vario Crosslinking; Peschke Meditrade GmcH/ 0.1% riboflavin 30 min	9mW/cm^2^ for 10 min	All eyes stabilised after treatment without any further signs of progression.	Max K: 48.89 ± 2.85	From 48.89 ± 2.85 to 49.21 ± 3.15 (P = 0.956)	From 0.33 ± 0.18 to 0.37 ± 0.18 (LogMAR) (P = 0.649)	From 0.13 ± 0.10 to 0.15 ± 0.12 (LogMAR) (P = 0.616)	-
Ozgurhan *et al*, 2014 (paeds) [65]	Retrospective interventional case series/ Keratoconus	44	24	↑ Kmax of ≥ 1D, ↑ astigmatism by ≥ 1D or ↑ MRSE of 0.50D/ 3 months	Avedro KXL/ 0.1% riboflavin 15 min	30mW/cm^2^ for 4 min	Improved UDVA and CDVA. Flat K value and steep K value decreased.	Max K: 50.6 ± 4.2	From 50.6 ± 4.2 to 50.1 ± 4.0 (P < 0.001)	From 0.52 ± 0.36 to 0.39 ± 0.26 (LogMAR) (P = 0.002)	From 0.38 ± 0.24 to 0.30 ± 0.20 (LogMAR) (P < 0.001)	SE: From -5.45 ± 2.99 to 5.27 ± 2.91 (P = 0.205)
Moramarco *et al*, 2015 [66]	Retrospective case series/ Keratoconus	60	1	Δ in corneal curvature in the cone area of ≥ 1.0 D or thinning of > 10μm in minimal pachymetry in 2 consecutive topography maps/ 6 months	Avedro KXL I/ 0.1% riboflavin with 1% HPMC 10 min	Pulsed: 30mW/cm^2^ 8 min Continuous: 30mW/cm^2^ 4 min	Pulsed accelerated CXL had a significantly deeper demarcation line as compared to continuous light exposure.	Max K (pulsed): 47 ± 6 Max K (continuous): 48.6 ± 3.8 (P > 0.05)	-	-	-	-

*UV* = Ultraviolet *pre-op* = pre-operative *UCVA* = Uncorrected Visual Acuity *BCVA* = Best Corrected Visual Acuity *UDVA* = Uncorrected Distance Visual Acuity

*CDVA* = Corrected Distance Visual Acuity *Kmax* = maximum keratometry *Kmean* = mean keratometry *K* = Keratometry *SE* = Spherical Equivalent *CXL* = cross-linking

*cyl* = cylinder *D* = Diopters *MRSE* = Manifest Refractive Spherical Equivalent *max* = maximum *paeds* = paediatric

**Table 5:** Summary of outcomes for cross-linking in infectious keratitis.

**Study**	**Study design**	**Indication**	**No. of Eyes**	**Follow-up, month**	**Other treatment**	**Findings**
Iseli *et al*, 2008 [114]	Prospective case series	Infectious keratitis unresponsive to antibiotics	5	1-9	Topical and systemic antibiotic therapy	In all cases, progression of corneal melting was halted. Emergency keratoplasty was not required in any of the cases.
Micelli Ferrari *et al*, 2009 [115]	Case report	Bacterial keratitis caused by Gram negative E. coli	1	1	Topical and systemic antimicrobial therapy	Corneal edema almost completely resolved, corneal ulceration healed after 1 month
Makdoumi *et al*, 2010 [116]	Prospective case series	Infectious keratitis associated with corneal melting	7	1-6	Topical antibiotics (all except 1)	Corneal melting arrested and complete epithelialisation was achieved in all cases.
Moren *et al*, 2010 [117]	Case report	Suspected acanthamoeba keratitis	1	9	Broad-spectrum antibiotics	Rapid decrease of pain and necrotic material. Corneal reepithelialisation started within a few days and completed within 1 month. Complete wound healing after 2 months. BCVA improved from 20/1000 to 20/30 after 9 months.
Khan *et al*, 2011 [118]	Interventional case series	Acanthamoeba keratitis unresponsive to treatment	3	2	Multidrug conventional therapy	Rapid reduction in symptoms and decreased ulcer size after the first treatment session. Progress of improvement slowed after 1 to 3 weeks but renewed after the second application. Ulcers closed within 3 to 7 weeks of first application. In 2 patients, penetrating keratoplasty was subsequently performed for residual dense corneal scars.
Anwar *et al*, 2011 [119]	Retrospective case reports	Infective keratitis unresponsive to antimicrobial therapy	2	-	Antimicrobial therapy	Rapid resolution of infective keratitis, leaving residual stromal scarring. 1 patient required penetrating keratoplasty for residual dense corneal scars.
Makdoumi *et al*, 2012 [120]	Prospective non-randomised study	Bacterial keratitis	16	-	Antibiotics only given for 2 out of 16 eyes	All eyes responded to photochemical treatment. Improved symptoms, reduced inflammation. Epithelial healing achieved. One patient required human amniotic membrane transplant.
Price *et al*, 2012 [125]	Prospective, dual-center, interventional case series	Infective keratitis (bacterial, fungal, protozoan, viral)	40	-	Standard antibiotic treatment, 7 patients had previous keratoplasty	Keratitis did not resolve in 6 cases and penetrating keratoplasty was needed. CXL should be avoided in eyes with prior herpes simplex. CXL appeared most effective when infection depth was limited. Success higher for bacterial than fungal infections.
Kymionis *et al*, 2012 [121]	Case report	Intractable post-laser keratitis due to atypical mycobacteria	1	3	Maximum antibiotic therapy	All infiltrates and stromal edema resolved after 1 week. UDVA improved from counting fingers at 3 meters to 20/35.
Li *et al*, 2013 [122]	Prospective case series	Fungal keratitis unresponsive to treatment	8	-	Topical antibiotics	No complications noted. Hypopyon disappeared in all cases between 3 to 11 days after CXL. Healing of corneal epithelium and ulcer was achieved between 3 and 8 days after CXL.
Arance-Gil *et al*, 2014 [131]	Case report	Acanthamoeba keratitis unresponsive to medical treatment	1	9	Medical treatment	After CXL, symptoms and corneal appearance improved significantly but the ulcer did not heal completely. Patient required amniotic membrane transplantation and penetrating keratoplasty.
Saglk *et al*, 2013 [123]	Case report	Suspected fungal keratitis unresponsive to treatment	1	6	Extensive medical treatment	Epithelial defect disappeared and stromal infiltrate stayed inactive from 1 week to 6 months after the second treatment.
Shetty *et al*, 2014 [127]	Prospective case series	Microbial keratitis (bacterial and fungal)	15	-	Antibiotics / antifungals	6/9 patients with bacterial keratitis and 3/6 patients with fungal keratitis resolved after CXL treatment. Patients with deep stromal keratitis or endothelial plaque failed to resolve.
Tabibian *et al*, 2014 [124]	Case report	Atypical fungal keratitis (Aureobasidium pullulans)	1	-	None	Corneal epithelium closed completely within 3 days and infiltrate was completely eradicated.
Said *et al*, 2014 [128]	Prospective clinical trial	Infectious keratitis with corneal melting (bacterial, fungal, amoebic)	40; 21 case, 19 control	-	Case: Antibiotics + CXL Control: Antibiotics only	Average healing time was 39.76 +/- 18.22 (PACK-CXL) and 46.05 +/- 27.44 (control). CDVA after healing was 1.64 +/- 0.62 (PACK-CXL) and 1.67 +/- 0.48 (control). The PACK-CXL group had a bigger corneal ulceration width and length.
Vajpayee *et al*, 2015 [129]	Retrospective case-file analysis	Moderate mycotic keratitis	41; 20 case, 21 control	-	Case: Antibiotics + CXL Control: Antibiotics only	Average healing time and final BCVA were similar in both groups. The additional CXL treatment did not have any advantage over medical treatment.
Uddaraju *et al*, 2015 [130]	Randomised clinical trial	Nonresolving deep stromal fungal keratitis	13; 6 case, 7 control	-	Case: Antibiotics + CXL Control: Antibiotics only	The trial was stopped due to a marked difference in the rate of perforation between the 2 groups. The CXL group had a significantly higher rate of perforation.
Bamdad *et al*, 2015 [132]	Prospective randomised clinical study	Moderate bacterial corneal ulcers	32; 16 case, 16 control	0.5	Case: Antibiotics + CXL Control: Antibiotics only	Mean treatment duration was 17.2 +/- 4.1 days in the case group and 24.7 +/- 5.5 days in the control group. Epithelial defects were smaller in the case group at 7 and 14 days.

*BCVA* = Best Corrected Visual Acuity *UDVA* = Uncorrected Distance Visual Acuity *CXL* = Cross-Linking *PACK-CXL* = Photo Activated Chromophore for keratitis
